# Adaptive control for memristive system via compensatory controller and Chebyshev neural network

**DOI:** 10.1038/s41598-024-61593-3

**Published:** 2024-06-09

**Authors:** Shaofu Wang

**Affiliations:** School of Information and Management, Guangxi Medical University, Nanning, 530021 People’s Republic of China

**Keywords:** Mathematics and computing, Physics

## Abstract

In this paper, based on linear matrix inequality technique, a simple controller and a compensatory controller are designed. It can track arbitrary fixed points and any periodic orbits. In addition, a synchronization control method via Chebyshev neural network with external disturbances is proposed. An adaptive controller is given. The Chebyshev neural network is used to approximate the uncertain nonlinear function and the adaptive law is used to adjust the corresponding parameters in the system. Taking a 4D memristive chaotic system as examples, the results are in consistent with the simulations. From a framework and control theoretical point of view, the proposed synchronization approach via compensation controller and Chebyshev neural network is firstly presented. From an application point of view, the proposed scheme can simplify the complexity of controller design. It is promising in many applications for mem-systems as secure communications and neural networks.

## Introduction

The brain is composed of neurons with different structures and functions, and studying the structure and function of these neurons is a significant and challenging frontier hot spot^1^. Through the continuous exploration of scholars, the neural network has been formed to study brain science based on the structure and function of neurons^2^. Neural network simulates neuronal activities with mathematical models, which can realize human brain's thinking, perception and other behaviors^3^. In the past few decades, neural networks have received extensive attention from scholars and have been applied in many fields, such as secure communication^4^, fault detection^5^, intelligent control^6^, machine learning^7^ and deep learning^8^. In the general neural network model, the resistors act as biological synapses, but ordinary resistors can not meet the needs of memory, and memristors can achieve this function. The memristor was proposed by Professor Chua^9^ in 1971 and implemented by Strukov and Snidein^10^ in 2008. Memristor neural networks are characterized by plastic response, non-volatility, nanoscale size, low energy consumption, storability and easy integration^11^. In addition, among the many dynamic behaviors of neural networks, tracking and synchronization are very meaningful research content^12^. Tracking and synchronization mean that related neural network nodes achieve the same dynamic behavior. Pinning synchronization can be divided into pinning synchronization^13^, sliding mode synchronization^14^ and exponential synchronization^15^ and fixed/prescribed-time synchronization^16^, etc. However, it is often impossible to obtain the exact initial state value of the system in practical application. In order to solve this problem, Cheng and Tang^17^ proposed the finite-time and fixed-time synchronization of delayed memory neural networks based on adaptive aperiodic intermittent adjustment strategy in 2023. Compared with the uncertainty of infinite rest time in asymptotic and exponential synchronization theory and the initial value dependence of finite rest time in finite time synchronization theory, the certainty and independence of finite and fixed-time synchronization theory have been greatly improved. The practical applications of neural network tracking and synchronization theory, such as tracking control and secure communication, have received extensive attention and in-depth research^18–35^.

However, for the above works, the controller design is only for a specific chaotic networks and does not have universality. The adaptive control of memristive system based on linear matrix inequalities technique and Chebyshev neural network has rarely been reported.

Based on Lyapunov stability theory, a suitable nonlinear adaptive controller and compensation controller are designed in this paper, and the synchronization problem of a class of memristive systems and neural networks is studied. The main highlights and contributions are summarized as follows: (1) Different from the traditional stability theory, the compensatory controller and adaptive controller used in this paper require fewer parameters, reduce energy consumption, and have better economic mechanism. (2) Adding memristive components that are highly similar to biological synapses to the traditional neural network model can better simulate the human brain. (3) Based on linear matrix inequality technique, a simple controller and a compensatory controller and the update law are designed. (4) A novel synchronization method via Chebyshev neural network is proposed, then, taking this memristve system with external disturbances as an example, the simulation results verify the effectiveness of the proposed method.

## Tracking control method

The memristive system is described as

1 $$ \dot{x} = Ax + F(x), $$

where $x \in R^{n}$ is the state variable, $A \in R^{n \times n}$ is the coefficient matrix, $F(x) \in R^{n}$ is the nonlinear function which satisfies the following Lipschitz conditions:

2 $$ \left\| {F(x) - F(\hat{x})} \right\| \le L\left\| {x - \hat{x}} \right\|, $$

where $L > 0$, $\hat{x} = [\hat{x}_{1} ,\hat{x}_{2} , \ldots ,\hat{x}_{n} ]^{T}$ denotes Euclidean norm.

**Figure 1:**
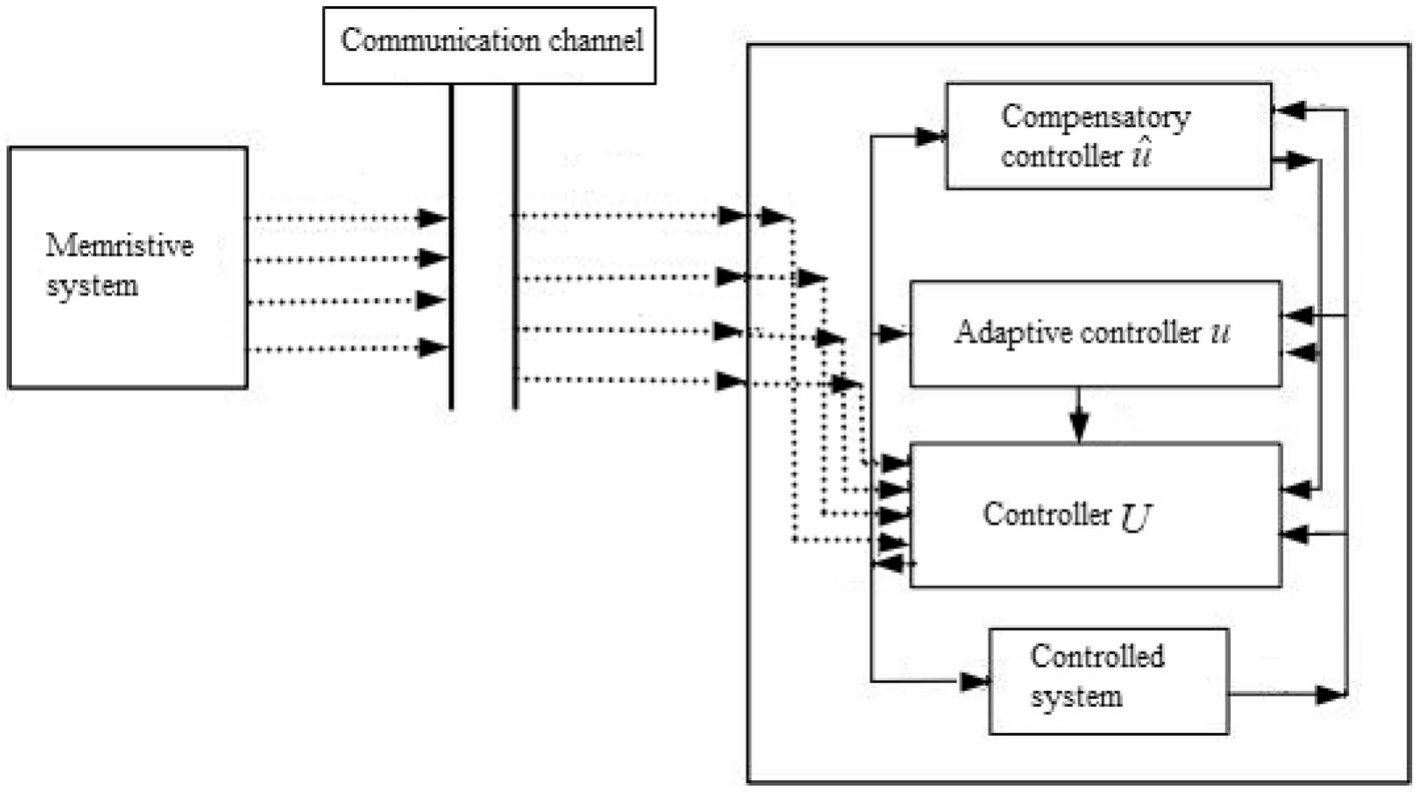
The structure of the proposed adaptive controlled system.

The structure of the proposed adaptive controlled memristive system is shown in Fig. 1. Assuming the reference signal $\hat{x} = [\hat{x}_{1} ,\hat{x}_{2} , \ldots ,\hat{x}_{n} ]^{T}$ and a tracking controller $U$ to (1), one has

3 $$ \dot{x} = Ax + F(x) + U. $$

where $$U = [U_{1} ,U_{2} , \ldots ,U_{n} ]^{T}$$.

In order to realize $x$ to track the reference signal, it needs the next steps.

Applying a compensatory controller $\hat{u} = [\hat{u}_{1} ,\hat{u}_{2} , \ldots ,\hat{u}_{n} ]^{T}$ to (1), one obtains

4 $$ \dot{\hat{x}} = A\hat{x} + F(\hat{x}) + \hat{u}. $$

Defining the error $e_{i} = \hat{x}_{i} - x_{i}$, $(i = 1,2, \ldots ,n)$, let

5 $$ \hat{u} - U = - u, $$

where $u$ is the adaptive controller, Subtracting (3) from (4). One has

6 $$ \dot{e} = Ae + F(\hat{x}) - F(x) - u. $$

### Theorem 1

Under the nonlinear function $F(\hat{x}) - F(x)$ of (6) satisfies the Lipschitz condition (2), if the controller and update law are designed, respectively, as

7 $$ u = \Gamma e + k\left\| e \right\|\frac{\Theta e}{{\left\| {\Theta e} \right\|}}, $$

8 $$ \dot{k} = \mu \left\| {\Theta e} \right\|\left\| e \right\|. $$

where $q \in R^{ + }$, $\mu \in R^{ + }$, $\Gamma \in R^{n \times n}$, $\Theta \in R^{n \times n}$ is a positive definite symmetric matrix which satisfies the following linear matrix inequality :

9 $$ (A - \Gamma )^{T} \Theta + \Theta (A - \Gamma )^{T} < 0, $$

The error $e(t)$ is globally asymptotically stable to zero under the action of the controller (7) and update law (8).

### Proof

Define Lyapunov function as

10 $$ V = e^{T} \Theta e + \frac{1}{\mu }\left( {k - L} \right)^{2} , $$

Since $\Theta$ is a positive definite matrix, $V$ is also positive definite. One has

11 $$\begin{aligned} \dot{V} &= \dot{e}^{T} \Theta e + e^{T} \Theta \dot{e} + \frac{2}{\mu }\dot{k}\left( {k - L} \right) \\ & = 2\{ e^{T} A^{T} + [F(\hat{x}) - F(x) - u]^{T} \} \Theta e + \frac{2}{\mu }\dot{k}\left( {k - L} \right)  \\ & = 2e^{T} A^{T} \Theta e + 2[F(\hat{x}) - F(x)]^{T} \Theta e - 2\left[ {e^{T} \Gamma^{T} + \frac{k\left\| e \right\|}{{\left\| {\Theta e} \right\|}}e^{T} \Theta^{T} } \right]\Theta e + 2(k - L)\left\| {\Theta e} \right\|\left\| e \right\|  \\ & \le 2e^{T} A^{T} \Theta e - 2e^{T} \Gamma^{T} \Theta e + 2L\left\| {\Theta e} \right\|\left\| e \right\| - 2k\left\| {\Theta e} \right\|\left\| e \right\| + 2k\left\| {\Theta e} \right\|\left\| e \right\| - 2L\left\| {\Theta e} \right\|\left\| e \right\|   \\ &= 2e^{T} \left( {A^{T} \Theta - \Gamma^{T} \Theta } \right)e \\ & = e^{T} \left[ {(A - \Gamma )^{T} \Theta + \Theta (A - \Gamma )^{T} } \right]e, \end{aligned}$$

One has

12 $$ V \le e^{T} [(A - \Gamma )^{T} \Theta + \Theta (A - \Gamma )^{T} ]e, $$

According to (9), one has

13 $$ \dot{V} \le 0. $$

Therefore, the error (6) is globally asymptotically stable at zero. The proof is completed.

### Remark 1

The linear matrix inequality Eq. (9) holds that all the eigenvalues of the matrix $A - \Gamma$ are negative. And the controller $u$ is derived by selecting a matrix such that all eigenvalues of the matrix $A - \Gamma$ are negative, and a positive definite symmetric matrix $\Lambda$ is arbitrarily defined. The positive definite symmetric matrix $\Theta$ can be obtained, so that the adaptive controller $u$ can be determined by (7). The expression of the compensatory controller $\hat{u}$ can be easily determined by substituting the reference signal into the system.

## Synchronization control via Chebyshev neural network

The driven system is described as

14 $$ \dot{x}(t) = [A + \Delta A(t)]x + f(x) + \Delta f(x,t) + d_{m} (t), $$

The corresponding response system is given as

15 $$ \dot{y}(t) = [B + \Delta B(t)]y + g(y) + \Delta g(y,t) + d_{s} (t) + u(t), $$

where $x = [x_{1} ,x_{2} , \cdots ,x_{n} ]^{T}$, $y = [y_{1} ,y_{2} , \cdots ,y_{n} ]^{T}$. $A$ and $B$ are constant matrice, $\Delta A$ and $\Delta B$ are the perturbations, $f$ and $g$ are nonlinear functions, $\Delta f$ and $\Delta g$ are uncertainties, $d_{m} (t)$ and $d_{s} (t)$ are external disturbances and the synchronization error $e = y - x$. One has

16 $$ \dot{e} = By - Ax + \Delta B(t)y - \Delta A(t)x + g(y) - f(x) + \Delta g(y,t) - \Delta f(x,t) + d_{s} (t) - d_{m} (t) + u(t). $$

**Figure 2:**
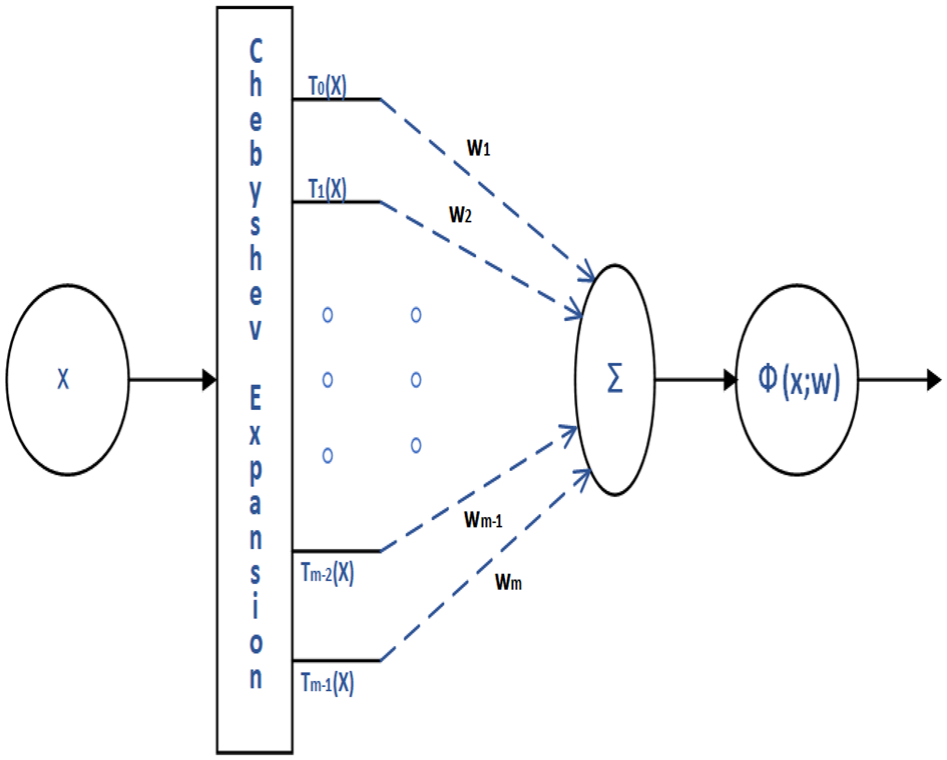
The structure of Chebyshev neural network.

The structure of Chebyshev neural network is depicted as Fig. 2.

### Lemma 1

If $\mathop {\lim }\limits_{t \to \infty } \xi (t) \to \Theta$ and $\dot{\xi }(t)$ is bounded, then, $\mathop {\lim }\limits_{t \to \infty } \xi (t) \to 0$.

### Theorem 2

Considering the interval $[ - 1,1]$, Chebyshev polynomials, which are given as

17 $$ T_{i + 1} (x) = 2xT_{i} (x) - T_{i - 1} (x), \quad i = 1.2. \ldots ,m - 1. $$

and the function $\phi (x)$ of Chebyshev neural network is considered as

18 $$ \phi_{LP} (x) = \sum\limits_{i = 1}^{m} {w_{i} } T_{i} (x) = W^{T} T, $$

where $$W = [w_{0} ,w_{1} , \ldots ,w_{m} ]^{T}$$, $$T = [T_{0} ,T_{1} , \ldots ,T_{m} ]^{T}$$.

And $\phi (x)$ can be written as

19 $$ \phi (x) = W^{T} T + \varepsilon , $$

The controller can be designed as

20 $$ u = - \sigma E - \hat{f} - u_{r} . $$

The compensatory controller is designed as

21 $$ u_{r} = \lambda {\text{sgn}} (E^{T} \Theta M), $$

where $\sigma > 0$ is the matrix of feedback gains, $\Theta$ is a symmetric positive definite matrix as (9). $\lambda$ is a constant.$M = I$,$\hat{f} = T\hat{\eta }$ is the uncertain estimator using Chebyshev neural network, suppose that the first five polynomials are chosen as follow:

22 $$ T = \left[ {\begin{array}{*{20}c} {T_{0} } & 0 & 0 & 0 \\ 0 & {T_{0} } & 0 & 0 \\ 0 & 0 & {T_{0} } & 0 \\ 0 & 0 & 0 & {T_{0} } \\ \end{array} } \right], $$

where $$T_{0} = [1,T_{1} ,T_{2} ,T_{3} ,T_{4} ]$$,$$\hat{\eta } = [\hat{\eta }_{1}^{T} ,\hat{\eta }_{2}^{T} ,\hat{\eta }_{3}^{T} ,\hat{\eta }_{4}^{T} ]$$, $$\hat{\eta }_{i} = [w_{0i} ,w_{1i} ,w_{2i} ,w_{3i} ,w_{4i} ]^{T}$$, and

23 $$ T_{1} = x, \; T_{2} = 2x^{2} - 1,\; T_{3} = 4x^{3} - 3x, \; T_{4} = 8x^{4} - 8x^{2} + 1. $$

$w_{0i}$, $w_{1i}$, …,$w_{4i}$ denote the coefficients of Chebyshev neural network to be estimated.

Then, the synchronization between (14) and (15) can be achieved.

### Proof

Define Lyapunov function as

24 $$ V = \frac{1}{2}E^{T} \Theta E + \frac{1}{2\gamma }\overline{\eta }^{T} \overline{\eta }, $$

where $\Theta$ is a symmetric positive definite matrix as (9). $\overline{\eta } = \eta^{*} - \hat{\eta }$,$\gamma$ is a positive constant. The time derivative of $V$ is

25 $$ \dot{V} = - \frac{1}{2}(\dot{E}^{T} \Theta E + E^{T} \Theta \dot{E}) - \frac{1}{\gamma }\overline{\eta }^{T} \dot{\hat{\eta }}, $$

One has

26 $$ \dot{V} = - \frac{1}{2}E^{T} \Gamma E + \overline{\eta }^{T} \phi^{T} M^{T} \Theta E + E^{T} \Theta M(\vartheta - u_{r} ) - \frac{1}{\gamma }\overline{\eta }^{T} \dot{\hat{\eta }}, $$

where $$\left\| {\vartheta (t)} \right\| \le \lambda$$, let

27 $$ \dot{\hat{\eta }} = \gamma \phi^{T} M^{T} \Theta E, $$

Then, Eq. (26) can be rewritten as

28 $$ \dot{V} = - \frac{1}{2}E^{T} \Gamma E + E^{T} \Theta M[\vartheta (t) - u_{r} ], $$

If one assumes the compensatory controller $u_{r}$ satisfies

29 $$ E^{T} \Theta M[\vartheta (t) - u_{r} ] \le 0, $$

Substituting (21) into (29), one has

30 $$ E^{T} \Theta \vartheta (t) - E^{T} \Theta \lambda {\text{sgn}} (E^{T} \Theta ) \le 0, $$

One has

31 $$ \left\| {E^{T} \Theta M} \right\|\left\| {\vartheta (t)} \right\| - \lambda \left\| {E^{T} \Theta M} \right\| \le 0, $$

One has

32 $$ \left\| {E^{T} \Theta M} \right\|\left[ {\left\| {\vartheta (t)} \right\| - \lambda } \right] \le 0, $$

According to Lemma 1, assuming that $\left\| {\vartheta (t)} \right\| \le \lambda$, one may get

33 $$ \dot{V} \le 0. $$

Therefore, Theorem 2 is completely proved.

## Model of memristive system

**Figure 3:**
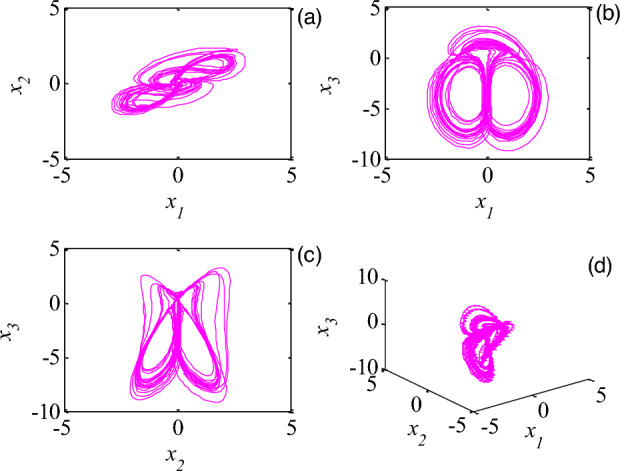
The projection phase of memristive system (34), (**a**) $x,y$, (**b**) $x,z$, (**c**) $y,z$, (**d**) $x,y,z$.

A memristive system is described as:

34 $$ \left\{ {\begin{array}{*{20}l} {\dot{x} = ax + by + yz} \hfill \\ {\dot{y} = - xz + yz - cF(y,\omega )} \hfill \\ {\dot{z} = - z - dxy + e} \hfill \\ {\dot{\omega } = y} \hfill \\ \end{array} } \right., $$

where

35 $$ F = (1 + 0.25z^{2} - 0.0002z^{4} )y, $$

when $a = 0.5$, $b = 3$, $c = 0.1$, $d = 6$, $e = 1$, initial conditions $x(0) = - 1$, $y(0) = - 1.5$, $z(0) = - 0.1$,$\omega (0) = - 0.5$, the phase diagram of system (34) are plotted in Fig. 3.

## Results and discussion

Taking the memristive system (34) as an example, rewrite it as

36 $$ \left[ {\begin{array}{*{20}c} {\dot{x}_{1} } \\ {\dot{x}_{2} } \\ {\dot{x}_{3} } \\ {\dot{x}_{4} } \\ \end{array} } \right] = \left[ {\begin{array}{*{20}c} {0.5} & 3 & 0 & 0 \\ 0 & { - 0.1} & 0 & 0 \\ 0 & 0 & { - 1} & 0 \\ 0 & 1 & 0 & 0 \\ \end{array} } \right]\left[ {\begin{array}{*{20}l} {x_{1} } \\ {x_{2} } \\ {x_{3} } \\ {x_{4} } \\ \end{array} } \right] + \left[ {\begin{array}{*{20}c} {x_{2} x_{3} } \\ { - x_{1} x_{3} + x_{2} x_{3} - 0.025x_{2} x_{4}^{2} + 0.0002x_{2} x_{4}^{4} } \\ { - 6x_{1} x_{2} } \\ 0 \\ \end{array} } \right], $$

**Figure 4:**
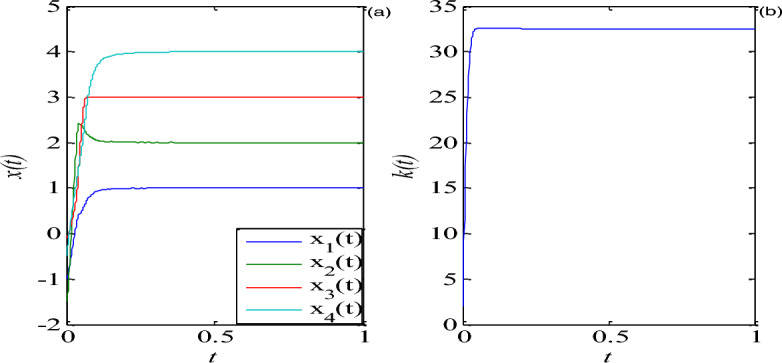
The tracking control of fixed-point $P$. (**a**) Time domain response, (**b**) the parameter $k(t)$.
II. The tracking control of periodic signal
  Assume the reference signal $r = [\sin t,\cos t,2\sin t,2\cos t]^{T}$, under the action of the compensatory controller, tracking controller, the update law and linear matrix inequality, the initial conditions of the controlled system are chosen as $\hat{x}_{1} (0),\hat{x}_{2} (0),\hat{x}_{3} (0),\hat{x}_{4} (0) = [ - 1,1.5, - 0.1, - 0.5]$, and $\mu = 2$, $k(0) = 20$, the time domain response and the adaptive parameter $k(t)$ for the controlled system (36) are presented in Fig. 5.

I. The tracking control of fixed-point $P(c_{1} ,c_{2} ,c_{3} ,c_{4} )$.
  Assume the reference signal $\hat{x} = [1,2,3,4][1,2,3,4]$, according to (6), the compensatory controller can be obtained as
  37 $$ \left\{ {\begin{array}{*{20}l} {\hat{u}_{1} = - 12.5} \hfill \\ {\hat{u}_{2} = - 2.2048} \hfill \\ {\hat{u}_{3} = 14} \hfill \\ {\hat{u}_{4} = - 2} \hfill \\ \end{array} } \right., $$
  Let $\Gamma = {\text{Diag}}10,0,0,10$, one has
  38 $$ A = \left[ {\begin{array}{*{20}c} {0.5} & 3 & 0 & 0 \\ 0 & { - 0.1} & 0 & 0 \\ 0 & 0 & { - 1} & 0 \\ 0 & 1 & 0 & 0 \\ \end{array} } \right], \;\;\;A - \Gamma = \left[ {\begin{array}{*{20}c} { - 9.5} & 3 & 0 & 0 \\ 0 & { - 0.1} & 0 & 0 \\ 0 & 0 & { - 1} & 0 \\ 0 & 1 & 0 & { - 10} \\ \end{array} } \right], $$
  The eigenvalues of the matrix $A - \Gamma$ can be obtained as follow
  39 $$ \lambda_{1} = - 9.50,\lambda_{2} = - 10.0,\lambda_{3} = - 0.10,\lambda_{4} = - 1.0, $$
  It can be seen that all the eigenvalues are negative, and the positive real symmetric matrix $\Lambda =$ Diag $(5,5,5,5)$ is used, according to linear matrix inequality (12), a positive symmetric matrix can be gotten as
  40 $$ \Theta = \left[ {\begin{array}{*{20}c} {2.7303} & {7.8125} & 0 & {0.7814} \\ {7.8125} & {25.0000} & 0 & {2.4752} \\ 0 & 0 & {2.5000} & 0 \\ {0.7814} & {2.4752} & 0 & {0.4975} \\ \end{array} } \right], $$
  According to Eqs. (7), (8) and (9), when $\hat{x}_{1} (0),\hat{x}_{2} (0),\hat{x}_{3} (0),\hat{x}_{4} (0) = [ - 1,1.5, - 0.1, - 0.5]$, $k(0) = 2$, $\mu = 2$, the time domain response and the adaptive parameter $k(t)$ for the controlled mem-system (5) are drawn in Fig. 4.

**Figure 5:**
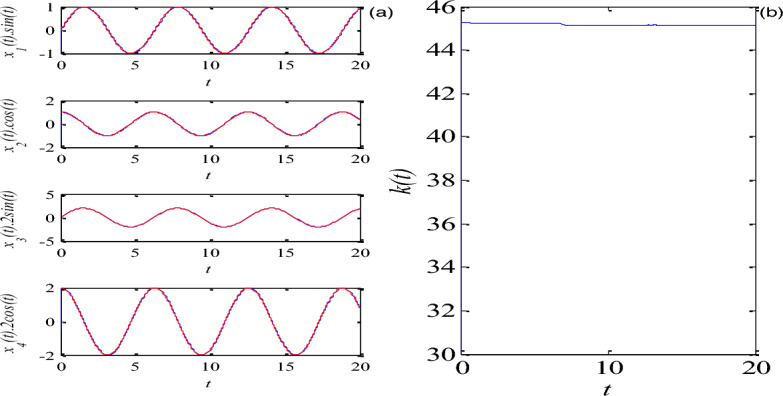
The tracking control of periodic signal, (**a**) time domain response, (**b**) the parameter $k(t)$.
  From Figs. 4 and 5, they can be observed that by using linear matrix inequality technique, the compensatory controller and tracking controller and update law can be designed, as time increases, all state variables of the controlled mem-system (5) can quickly track a fixed point and some periodic signals, and the adaptive update law $k(t)$ reaches a stable state.
III. The synchronization control via Chebyshev network
  Consider the identical driven and response system described in Eqs. (14) and (15) with the proposed memristive system (36) with external disturbances as an example, initial conditions: $$x(0) = [1,1,1,1]^{T}$$ and $$y(0) = [ - 1, - 1, - 1, - 1]^{T}$$,$$d_{m1} = 0.1\cos (t)$$, $$d_{m2} = 0.1\sin (t)$$, $$d_{m3} = 0.1\cos (t)$$,$$d_{m4} = 0.1\sin (t)$$, $$d_{s1} = 0.1\sin (2t)$$, $$w_{0i} (0) = w_{1i} (0) = w_{2i} (0) = w_{3i} (0) = w_{4i} (0) = 1$$,$$d_{s2} = 0.1\cos (2t)$$, $$d_{s3} = 0.1\sin (2t)$$, $$d_{s4} = 0.1\cos (2t)$$, $$\lambda = 5$$,$$\gamma = 0.1$$$$\sigma_{i} = 5$$, the unique input of Chebyshev neural network $$x = \sin (2\pi t)$$,the synchronization are illustrated in Fig. 6, it is clearly seen that the proposed controller is faster in reducing the time of synchronization errors. Moreover, the controller design is simpler. Chebyshev neural network can be used to approximate the uncertain nonlinear function and the adaptive law can be used to adjust its correspo- nding parameters in the system.

**Figure 6:**
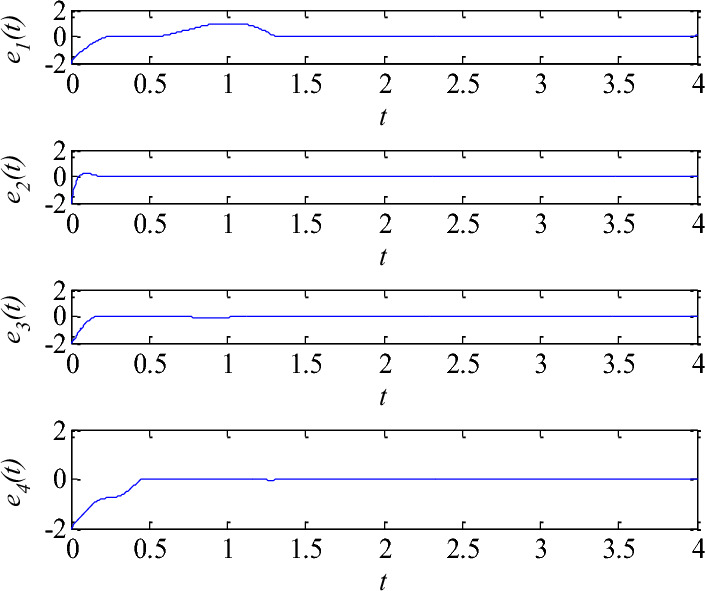
Synchronization control error $e(t)$.

## Conclusion

To sum up, a memristive system and the basic principle of tracking control method were introduced, and the stability theory about the tracking control was analyzed. The process was to track the signal with the chaotic variable information driven by the memristive system. Additionally, a synchronization method via Chebyshev neural network was proposed. Compared to previous control schemes, in this scheme, it has the following achievements: (1) The adaptive control methods of memristive system based on linear matrix inequality and Chebyshev neural network are firstly proposed. (2) It has wide control range. This scheme can stabilize the memristive system at arbitrary fixed points or any periodic orbit (see Figs. 4 and 5), the synchronization of two identical mem-systems with external disturbances can be fast achieved (see Fig. 6). The control process is simple and effective. Moreover, the structure of Chebyshev neural networks is simpler since there are fewer tuning parameters. it is particularly easy to implement in engineering. The fractional-order control method and their application in communication will be discussed in next work.

## Data Availability

Data is provided within the manuscript.
